# Genome-Wide Characterization and Analysis of Metallothionein Family Genes That Function in Metal Stress Tolerance in *Brassica napus* L.

**DOI:** 10.3390/ijms19082181

**Published:** 2018-07-26

**Authors:** Yu Pan, Meichen Zhu, Shuxian Wang, Guoqiang Ma, Xiaohu Huang, Cailin Qiao, Rui Wang, Xinfu Xu, Ying Liang, Kun Lu, Jiana Li, Cunmin Qu

**Affiliations:** 1 Key Laboratory of Horticulture Science for Southern Mountainous Regions, Ministry of Education, Southwest University, No. 2 Tiansheng Road, Beibei, Chongqing 400715, China; panyu1020@swu.edu.cn; 2 Academy of Agricultural Sciences, Southwest University, Chongqing 400715, China; zmc0809@email.swu.edu.cn (M.Z.); wsxsummer@email.swu.edu.cn (S.W.); mgqdw123456@email.swu.edu.cn (G.M.); hxh9305@email.swu.edu.cn (X.H.); qcl123@email.swu.edu.cn (C.Q.); ruiwang71@163.com (R.W.); xinfuxu@swu.edu.cn (X.X.); yliang@swu.edu.cn (Y.L.); drlukun@swu.edu.cn (K.L.); 3 Chongqing Rapeseed Engineering Research Center, College of Agronomy and Biotechnology, Southwest University, No. 2 Tiansheng Road, Beibei, Chongqing 400715, China

**Keywords:** metallothionein, *Brassica*, *Brassica napus*, As^3+^ stress

## Abstract

*Brassica* plants exhibit both high biomass productivity and high rates of heavy metal absorption. Metallothionein (MT) proteins are low molecular weight, cysteine-rich, metal-binding proteins that play crucial roles in protecting plants from heavy metal toxicity. However, to date, MT proteins have not been systematically characterized in *Brassica*. In this study, we identified 60 MTs from *Arabidopsis thaliana* and five *Brassica* species. All the MT family genes from Brassica are closely related to *Arabidopsis* MTs, encoding putative proteins that share similar functions within the same clades. Genome mapping analysis revealed high levels of synteny throughout the genome due to whole genome duplication and segmental duplication events. We analyzed the expression levels of 16 *Brassica napus* MTs (*BnaMTs*) by RNA-sequencing and real-time RT-PCR (RT-qPCR) analysis in plants under As^3+^ stress. These genes exhibited different expression patterns in various tissues. Our results suggest that *BnaMT3C* plays a key role in the response to As^3+^ stress in *B. napus*. This study provides insight into the phylogeny, origin, and evolution of MT family members in *Brassica*, laying the foundation for further studies of the roles of MT proteins in these important crops.

## 1. Introduction

Heavy metals are essential micronutrients for various physiological processes in plants. However, excess amounts of essential (copper, zinc) and non-essential metals (cadmium) are toxic to plants, as they inhibit plant growth, impair root development, and decrease chlorophyll contents, resulting in chlorosis [1,2]. Therefore, plants have evolved a suite of mechanisms involving the chelation and sequestration of heavy metals by various amino acids, organic acids, phytochelatins (PCs) and metallothioneins (MTs) [3,4]. These compounds play crucial roles in protecting plants from heavy metal toxicity [5,6,7,8,9].

MTs, low-molecular-weight proteins (7–10 kDa) with a high percentage of cysteine (Cys) residues [10,11], have been widely characterized in various prokaryotic and eukaryotic organisms. Plant MTs are classified into four types according to the arrangement of their Cys residues [12], including the *MT1*, *MT2*, *MT3*, and *MT4* subfamilies [10,13]. MTs play crucial roles in ion homeostasis and tolerance in plants. Seven functional MT genes have been isolated from the model plant *Arabidopsis thaliana*. Of these, *AtMT1a*, *AtMT2a*, *AtMT2b*, and *AtMT3* enhance plant tolerance of Cu ions, especially in leaves [14,15], as well as Cd tolerance in transformed yeast and fava bean (*Vicia faba*) guard cells [16,17]. *AtMT4* modulates Zn homeostasis in seeds and is highly expressed during the late stages of development [18]. Additionally, various *MT* genes show significantly different expression patterns in plants under heavy metal stress. For example, *MT2a* and *MT2b* genes are more highly expressed in the roots of the heavy metal hyperaccumulator *Noccaea caerulescens* than in those of *A. thaliana*, while *MT3* is more highly expressed in shoots of *N. caerulescens* than in the non-hyperaccumulator *Thlaspi arvense* [19,20]. *MT4* mRNAs are primarily expressed in ripening fruits and developing seeds [14]. Therefore, plant MTs are likely involved in many physiological processes, such as seed development and germination [18,21,22], fruit ripening [14], and root development [21,23].

*Brassica* plants are considered to be highly tolerant to heavy metals (e.g., Cd, Cu, Ni, Zn, Pb, and Se), making them ideal plants for studying metal accumulation in phytoremediation studies [24,25,26,27]. Indian mustard (*Brassica juncea)* is a high-biomass-producing crop with the potential to take up and accumulate heavy metals [1,23,27,28]. However, this plant accumulates Cd less effectively than other crops such as maize (*Zea mays*), rice (*Oryza sativa*), and sugar beet (*B. vulgaris*) when it is present at low concentrations in the soil [29]. Rapeseed (*B. napus*) has many advantages for this type of analysis due to its rapid growth, high biomass productivity and efficient heavy metal absorption, and it is therefore also widely used to investigate heavy metal tolerance [26,30,31,32]. Indeed, while heavy metal tolerance has been well studied in various *Brassica* species, the mechanisms that contribute to the tolerance of these plants to heavy metals remain unclear.

In the present study, we screened the draft genome sequences of *A. thaliana* and various *Brassica* species (*Brassica rapa*, *Brassica oleracea*, *B. napus*, *Brassica juncea* and *Brassica nigra*) for *MT* genes that participate in heavy metal detoxification. We identified 60 *MT* genes and performed a detailed analysis of their duplication patterns, classifications, and chromosomal distribution and motifs, as well as a phylogenetic analysis. Finally, we verified the differential expression profiles of selected rapeseed *MT* genes in different *B. napus* tissues at various developmental stages. We also investigated the expression patterns of *MT* genes in *B. napus* seedlings exposed to heavy metals. Our results provide important information about the origin and evolution of the *MT* gene family in *Brassica* and provide a basis for further studies of the functions of MT family proteins in rapeseed.

## 2. Results

### 2.1. Identification and Multiple Sequence Alignment of MT Family Genes

**Figure 1:**
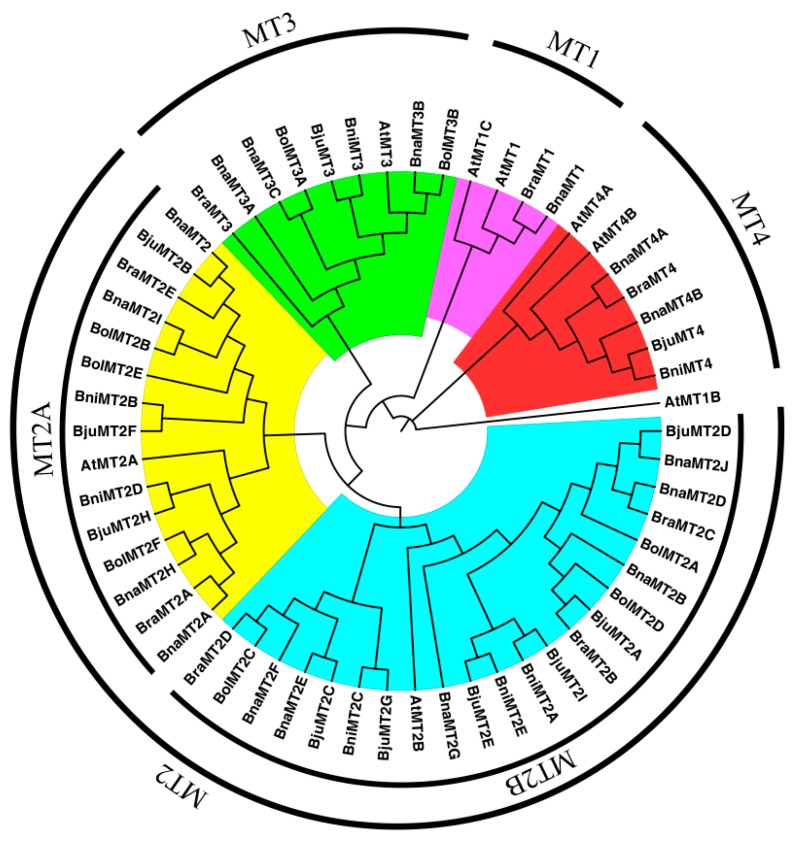
Neighbor-Joining (NJ) phylogenetic tree showed the relationships of *Metallothionein* (*MT*) family genes from *A. thaliana* and various *Brassica* species. The rooted neighbor-joining phylogenetic tree was constructed using MEGA6 and visualized using Figure Tree v1.4.2. The MTs were divided into four subfamilies (MT1–MT4), which are indicated by different colors. Organism name and gene accession numbers are shown in Table 1.

**Figure 2:**
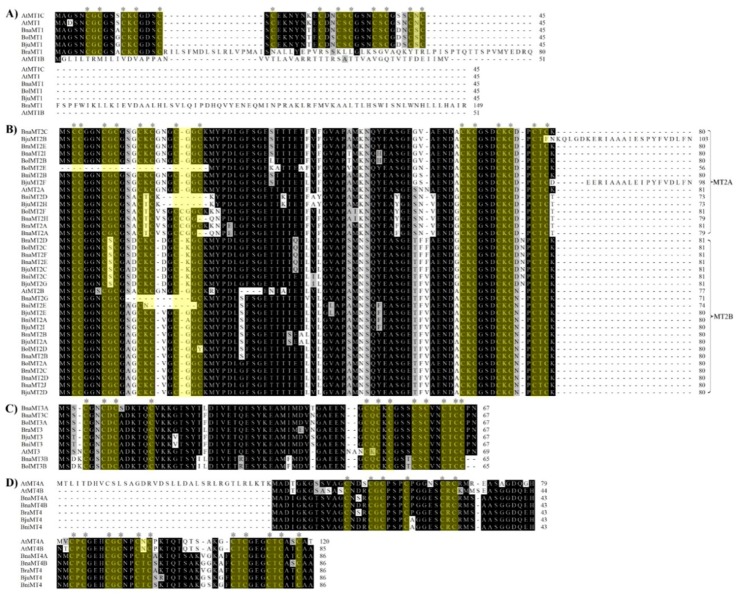
Alignment of MT protein sequences from *A. thaliana* and various *Brassica* species. Black and light gray shading indicate identical and conserved amino acid residues, respectively. (**A**) The MT1 protein sequences; (**B**) the MT2 protein sequences; (**C**) the MT3 protein sequences; (D) the MT4 protein sequences. The conserved cysteines regions are highlighted by asterisks and light yellow. The MTs were preliminarily classified by Cobbett and Goldsbrough reported [10]; detailed information is provided in Table 1.

Using the protein sequences of the *MT* family genes from the TAIR10 database (Table 1) as queries, we identified 60 *MT* genes in *A. thaliana* and various *Brassica* plants (*B. rapa*, *B. oleracea*, *B. napus*, *B. juncea* and *B. nigra*). These genes were classified into four subgroups (Figure 1, Table 1). Of these, seven were *MT1* subfamily members, five of which were identified from the corresponding genome databases, except *BolMT1* and *BjuMT1*. *BraMT1* has been reported in *B. rapa* with 45 amino-acid proteins [33]; the difference between these sequences requires further study. In addition, 37 were *MT2* subfamily members, encoding deduced proteins ranging from 56 to 103 amino acids in length; nine were *MT3* subfamily members, encoding proteins from 65 to 69 amino acids in length; and seven were *MT4* subfamily members, encoding proteins from 85 to 120 amino acids in length (Table 1). Of the *MT1* subfamily members, three homologs were identified in *A. thaliana*, while *BnaMT1* and *BraMT1* were identified in *B. napus* and *B. rapa*, respectively. No *MT1* subfamily members were found in *B*. *oleracea*, *B. juncea*, or *B. nigra*, whereas *BolMT1* and *BjuMT1* have been reported previously [13], indicating that genome gaps may still emerge in *B. oleracea*, and *B. juncea*. Six Cys-X-Cys motifs were equally distributed on the *N*- and *C*-termini of MT1 family proteins, except in the case of BraMT1 and AtMT1B (Figure 2A).

We identified 37 *MT2* subfamily genes in *A. thaliana* (*AtMT2A* and *AtMT2B*) and *Brassica* (five in *B. rapa*, six in *B. oleracea*, ten in *B. napus*, nine in *B. juncea*, and five in *B. nigra*), which were divided into the *MT2A* and *MT2B* subgroups (Figure 2B, Table 1), pointing to the extensive triplication and expansion of these genomes during their evolution in *Brassica* plants. Furthermore, one Cys–Cys and two Cys–X–Cys motifs were almost always present in the N-terminal regions of these proteins, and three Cys–X–Cys were almost always present in their C-terminal regions (Figure 2B). In addition, MT2 subfamily genes encode a deduced protein with the MSCCGGN/S sequence in their N-termini, which is consistent with previous findings [13,34]. Three variant regions were found in the MT2A subgroup and three in the MT2B subgroup (Figure 2B), which might be associated with their roles in metal tolerance.

We identified *MT3* subfamily genes, including one each in *A. thaliana*, *B. rapa*, *B. juncea*, and *B. nigra*, two in *B. oleracea* and three in *B. napus* (Figure 1, Table 1). The alignment of the MT3 amino acid sequences showed a completely conserved sequence, CXXCDCX_5_C, located in the N-terminus of each protein, and a highly conserved consensus sequence with eight Cys residues at the C-terminus (Figure 2C). In addition, 30–40 amino acids were detected in the Cys-poor linker region between the *N*- and *C*-terminal regions, in accordance with the MT2 subfamily (Figure 2B,C), pointing to a possible evolutionary relationship between the *MT2* and *MT3* family genes.

*MT4* subfamily genes, which are homologous to plant EC metallothionein-like genes, are different from *MT*1–*MT3* subfamily members. Two *MT4* subfamily members were found in *A. thaliana* and *B. napus*, one each in *B. rapa*, *B. juncea*, and *B. nigra*, and none in *B. oleracea* (Figure 1, Table 1). These proteins contain three Cys-poor linkers comprising 12–15 amino acids, as well as two Cys-rich regions with a highly conserved consensus sequence among them (Figure 2D).

### 2.2. Phylogenetic Analysis of MT Family Genes

Based on the multiple sequence alignment of the deduced MT1–MT4 proteins, it was found that Cys-rich regions are widely distributed among MT family proteins. These regions are characterized by conserved consensus sequences, with motifs such as Cys–G–Cys, Cys–K–Cys, and Cys–S–Cys (Figure 2). To investigate the evolutionary relationships among MT family genes from *A. thaliana* and various *Brassica* species, we constructed a NJ phylogenetic tree based on the alignment of MT domains. Based on the phylogenetic tree, the 58 MT domains were classified into four subfamilies (*MT1*, *MT2*, *MT*3, and *MT4*), except for *BolMT1* and *BjuMT1*, which were not annotated in the genome databases, and most genes were grouped with the *AtMTs* (Figure 2). *AtMT1B* represents the outgroup in the phylogenetic tree. In addition, the *MT2* subfamily was classified into two sister groups (Figure 1), *MT2A* and *MT2B*, which is highly consistent with the results of multiple sequence alignments of whole proteins (Figure 2). For example, two sister groups were also identified and found to contain eight and six *MT* family genes, respectively (Figure 2), which also contain highly conserved consensus sequences (Figure 1). These results will be helpful in identifying the functions of *MT* family genes via orthology analysis.

### 2.3. Genomic Structure and Conserved Motif Analysis of the MT Gene Family

**Figure 3:**
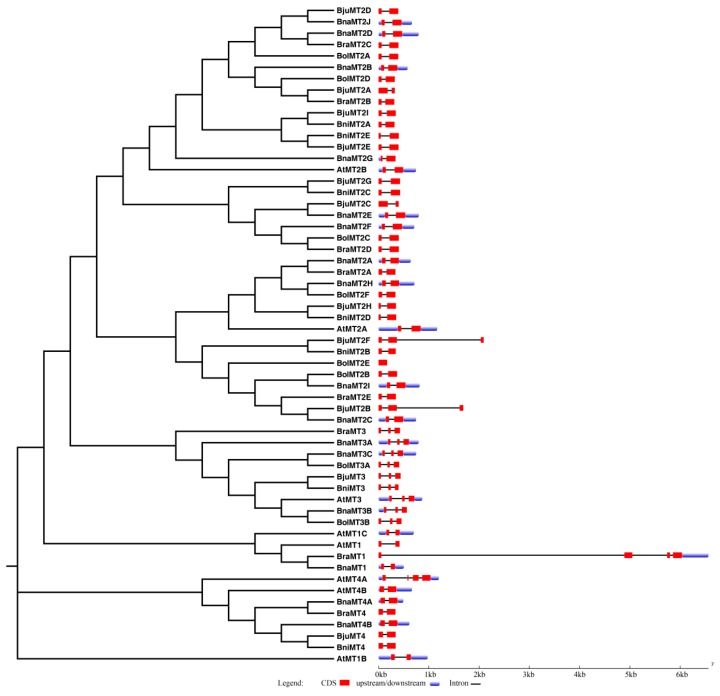
Phylogenetic relationships and genomic structures of the *MT* genes from *A. thaliana* and various *Brassica* species. The red boxes represent exons, solid lines represent introns (connecting two exons), and blue boxes represent untranslated regions (UTRs). The lengths of the *MT* genes are indicated by horizontal lines (kb).

We characterized the gene structures of the *MT* family genes by comparing the full-length CDS and the corresponding genomic DNA sequences using GSDS 2.0 (http://gsds.cbi.pku.edu.cn/index.php). Of the 58 *MT* genes, 44 contain a single intron with a highly conserved structure in each group, i.e., the *MT1*, *MT2* and *MT4* subfamilies. Additionally, *MT3* subfamily members contain two introns, which were also found in *BjuMT2B* and *BjuMT2F* (Figure 3). *BolMT2E* lacks an intron and belongs to the *MT2A* gene family, while *AtMT4A* and *BraMT1* contain three introns with distinct sizes (Figure 3). Most genes in the same subfamily exhibit similar exon–intron structures, but the genomic structures of *BjuMT2B* and *BjuMT2F* are similar to those of the *MT3* subfamily, providing further support for the evolutionary relationship and classification of the *MT* gene family members identified in this study.

**Figure 4:**
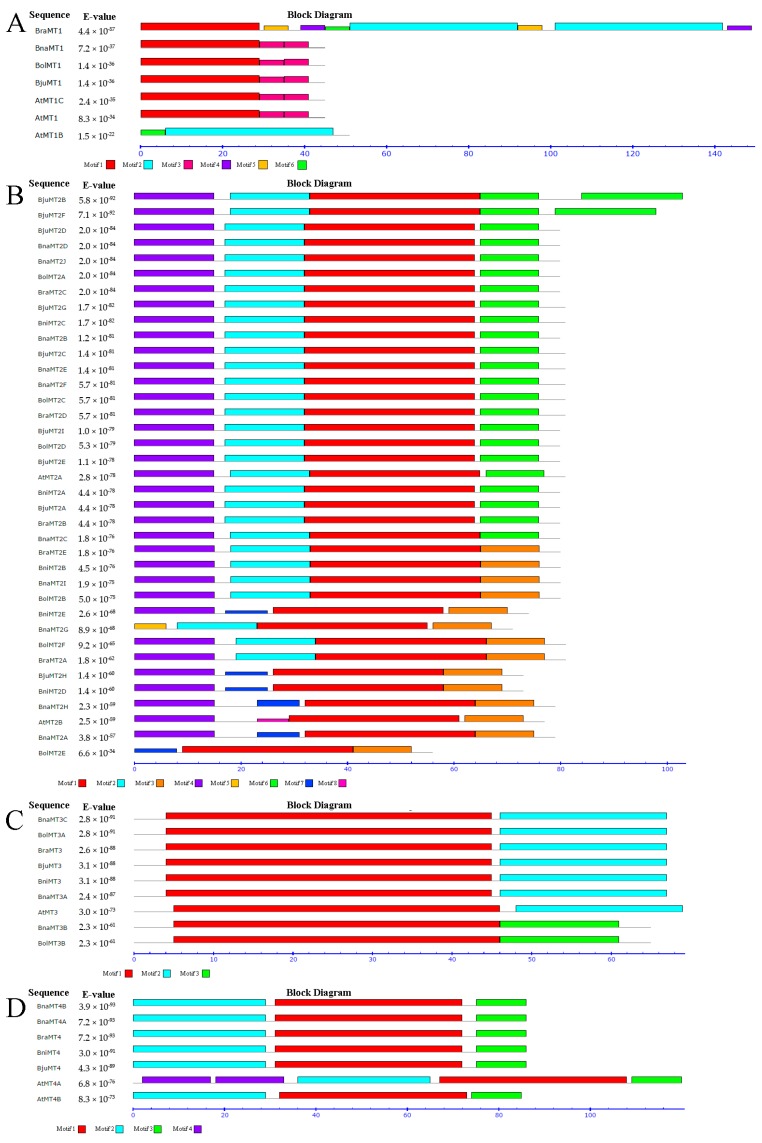
Putative conserved motifs in MT family proteins in various *Brassica* species identified using the MEME search tool. (**A**) the conserved motifs in MT1 family; (**B**) the conserved motifs in MT2 family; (**C**) the conserved motifs in MT3 family; (**D**) the conserved motifs in MT4 family. Different motifs are represented by different colors, and protein names and combined *p* values are shown on left side of this figure. The best possible matched motifs, their functional annotation, and motif width are shown in Figure S1.

Using MEME v4.12.0 (http://meme-suite.org/tools/meme), six, eight, three, and four conserved motifs were detected in the *MT1*, *MT2*, *MT3*, and *MT4* subfamilies, respectively, in *B. rapa*, *B. oleracea*, *B. napus*, *B. juncea*, and *B. nigra* (Figure 4A–D); the detailed structures of the motifs are shown in Figure S1A–D. All members of the *MT1* subfamily except for *AtMT1B* contain motif 1 (Figure 4A and Figure S1A). All members of the *MT2* subfamily contain motif 1, whereas all members of the *MT2A* subfamily contain motifs 2, 4, and 6, but motif 3 is found only in the *MT2B* subfamily members. The motifs in the *MT2B* subfamily members are more variable than those of the other *MTs* (Figure 4B and Figure S1B), pointing to the triplication and expansion of *Brassica* genomes. All nine genes in the *MT3* subfamily contain motif 1, while the *MT3A* subfamily genes contain motif 2 and the *MT3B* subfamily genes contain motif 3 (Figure 4C and Figure S1C). The *MT4* subfamily genes contain motifs 1, 2, and 3, indicating that these motifs are conserved among these genes (Figure 4D and Figure S1D). In summary, the same conserved motifs are widely found in paralogous/orthologous genes, suggesting that they might have similar functions at the protein level.

### 2.4. Chromosome Locations and Duplication of MT Genes in Brassica

**Figure 5:**
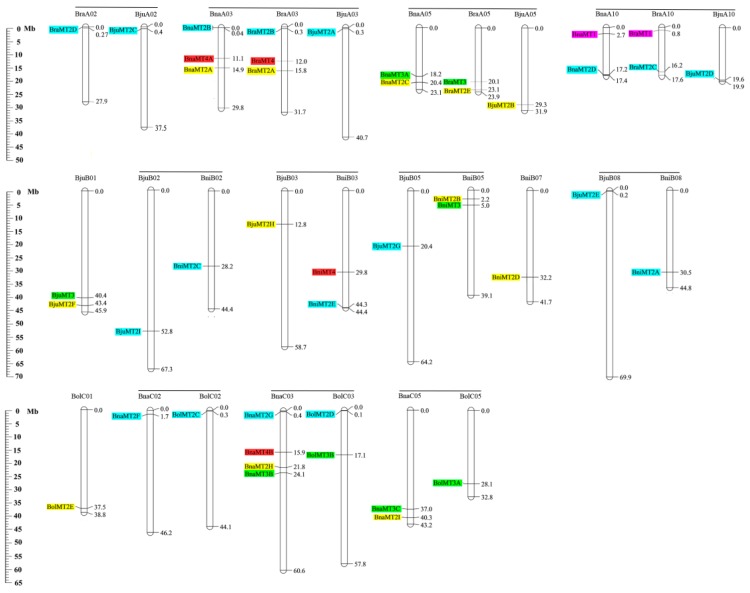
Chromosomal distribution and analysis of duplication events in *MT* family genes among *Brassica* species. Genes from the same subgroups are indicated by the same color, which is consistent with the corresponding family in the phylogenetic tree (Figure 1). The labels on the corresponding chromosomes indicate the names of the source organism and the subgenome. The scales indicate the sizes of various *Brassica* plant genomes (Mb). Bra, *B. rapa*; Bol, *B. oleracea*; Bni, *B. nigra*; Bna, *B. napus*; and Bju, *B. juncea*. The genes located on the scaffold are not shown in the Figure 5.

*Brassica* includes three diploid species, *B. rapa* (AA, 2*n* = 20), *B. oleracea* (CC, 2*n* = 18), and *B. nigra* (BB, 2*n* = 20) and three allotetraploid species, *B. napus* (AACC, 2*n* = 38), *B. juncea* (AABB, 2*n* = 36), and *B. carinata* (BBCC, 2*n* = 34), and the evolution and relationships between the members of *Brassica* can be well understood according to the U-triangle theory [35]. Five of these species have been completely sequenced, and their sequences are available in the *Brassica* database (BRAD) database. To identify the physical positions of the *MT* genes, we mapped them to the chromosomes in the corresponding *Brassica* species. The 43 *MT* genes are located on 27 chromosomes in the five *Brassica* species with available whole-genome sequences, including four chromosomes (BraA02, BraA03, BraA05, and BraA10) in *B. rapa*, four chromosomes (BolC01, BolC02, BolC03 and BolC05) in *B. oleracea* and five chromosomes in *B. nigra* (BniB02, BniB03, BniB05, BniB07 and BniB08) (Figure 5). Further, we detected high levels of synteny among *MT* family genes in these species. For example *BraMT2D* on chromosome BraA02, *BjuMT2C* on chromosome BjuA02, *BnaMT2F* on chromosome BnaC02, and *BolMT2C* on chromosome BolC02 are located near the top of the chromosomes and are classified into the same subgroups (Figure 2), suggesting that these genes might have undergone whole-genome duplication events during the evolutionary process and might have similar functions. However, some of these genes, e.g., *BnaMT4A* and *BraMT4* on chromosome A03, *BnaMT3A* and *BraMT3* on chromosome A05, and *BnaMT1* and *BraMT1* on chromosome A10 might have undergone segmental duplications (Figure 5). Finally, *BjuMT2H* and *BniMT2E* on chromosome B03 and *BjuMT2E* and *BniMT2A* on chromosome B08 might have undergone gene transposition (Figure 5). Taken together, these results shed light on the evolutionary patterns of these subfamilies among adjacent species.

### 2.5. Expression Profiles of BnaMT Family Genes in B. napus

**Figure 6:**
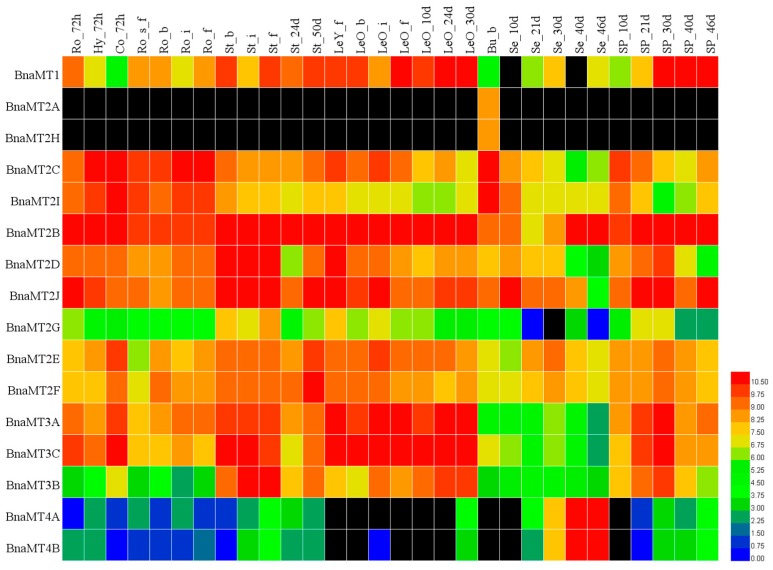
Heatmap of the expression profiles of *BnaMT* family genes in different tissues and organs. The abbreviations above the heatmap indicate the different tissues and organs/developmental stages of *B*. *napus* ZS11 (listed in Table S1). The expression data was gained from the RNA-seq data and shown as log2, as calculated by fragments per kilo base of exon model permillion (FPKM) values. Black boxes indicate that no expression was detected by RNA-seq analysis. The heatmap was generated using Heatmap Illustrator v1.0 (HemI v1.0, Huazhong University, Wuhan, China; http://hemi.biocuckoo.org/contact.php).

Based on the transcriptome sequencing datasets from *B. napus* ZS11 (BioProject ID PRJNA358784), we characterized the expression profiles of the *BnaMT* genes in eight different tissues, covering all stages of rapeseed development (Figure 6, Tables S1 and S2). Among the 16 *BnaMT* genes, *BnaMT1* was more highly expressed in the stems, leaves, and siliques 30 days after pollination than in other tissues (Figure 6). Among *MT2* genes, *BnaMT2A* and *BnaMT2H* were specifically expressed in buds; *BnaMT2C* and *BnaMT2I* were expressed at higher levels in roots, hypocotyls, cotyledons, and buds than in others tissues; *BnaMT2B*, *BnaMT2D*, and *BnaMT2J* were highly expressed throughout plant development, whereas *BnaMT2G* was expressed at low levels; and *BnaMT2E* and *BnaMT2F* were more highly expressed in stems and leaves than in other tissues (Figure 6). *BnaMT3A*, *BnaMT3B*, and *BnaMT3C* were more highly expressed in stems, leaves, and siliques before day 30 than in other tissues (Figure 6). Finally, *BnaMT4A* and *BnaMT4B* were mainly expressed in ripening seeds (Figure 6). The expression patterns of *MT* family genes correspond with the results of the phylogenetic analysis (Figure 2). For example, the expression patterns were similar for *BnaMT2A* and *BnaMT2H*, *BnaMT2C* and *BnaMT2I*, and *BnaMT2B*, *BnaMT2D*, and *BnaMT2J*, which were classified into the same sister groups.

### 2.6. Expression Analysis of BnaMT Genes in Response to Metal Treatment

**Figure 7:**
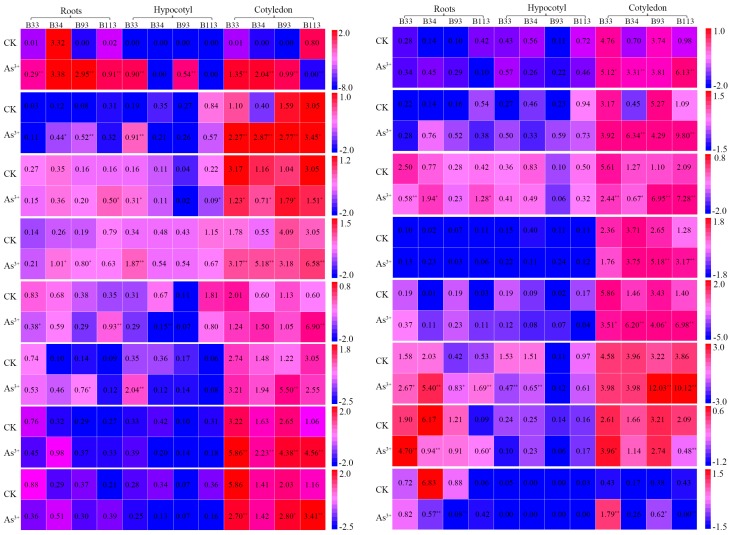
Expression analysis of *BnaMT* family genes in different tissues under control and As^3+^ treatment via real-time RT-PCR (RT-qPCR). Three biological replicates per sample were used for analysis, and three technical replicates were analyzed per biological replicate. Values represent the average of three biological replicates with three technical replicates of each tissue (Table S3). The expression data was gained from the real-time RT-PCR (RT-qPCR) analysis data and shown as log2 as calculated by average values normalized to that of the reference gene *BnACTIN7* (EV116054). *, ** indicates a significance level at 0.05 and 0.01, respectively. The heatmap was generated using Heatmap Illustrator v1.0 (HemI v1.0, Huazhong University, Wuhan, China; http://hemi.biocuckoo.org/contact.php).

MTs are the best-characterized heavy-metal-binding ligands in plants. To analyze the roles of *BnaMTs* in metal tolerance, we compared the expression profiles of *BnaMTs* in the roots, hypocotyls, and cotyledons of *B. napus* plants under As^3+^ stress versus normal conditions via real-time RT-PCR (RT-qPCR). Under normal conditions, the expression patterns of the *BnaMTs* were similar to the patterns identified by RNA-seq, with different expression profiles detected among different rapeseed varieties (Figure 7, Table S3). For example, *BnaMT2B*, *BnaMT2C*, *BnaMT2D*, and *BnaMT2J* were highly expressed in all tissues; *BnaMT1* and *BnaMT4B* were expressed at lower levels in roots, hypocotyls, and cotyledons; *BnaMT2A*, *BnaMT2F*, *BnaMT2G*, *BnaMT2H*, *BnaMT2I*, *BnaMT3A*, and *BnaMT3B* were expressed at lower levels in roots and hypocotyls than in cotyledons (Figure 7); and *BnaMT2A* and *BnaMT2H* did not exhibit tissue-specific expression in *B. napus* (Figure 6 and Figure 7). After As^3+^ treatment, all *BnaMT* genes were expressed at higher levels in roots than in hypocotyls but were expressed at the highest levels in cotyledons (Figure 7). For example, *BnaMT1* was upregulated by As^3+^ treatment, and *BnaMT2A*, *BnaMT2B*, *BnaMT2F*, *BnaMT2J*, and *BnaMT3B* were more significantly upregulated in cotyledons than in roots and hypocotyls (Figure 7). Importantly, *BnaMT3C* was more highly expressed in varieties B33 and B34 than in B93 and B113 (Figure 7).

## 3. Discussion

The high-affinity heavy metal chelators, PCs and MTs, play crucial roles in maintaining metal homeostasis during plant development [36,37,38,39,40,41]. Moreover, the *Brassica* plants had high biomass productivity and high levels of heavy metal absorption, as analyzed in *B. juncea* [1,23], *B. rapa* [33], and *B. napus* [42]. In addition, seven putative *MT* genes have been identified in *Arabidopsis* [15,16], but no comprehensive study of these genes has been reported. *Brassica* species, which were derived from a common ancestor, are ideal model systems for analyzing polyploid evolution and genome duplication [43]. Many analyses have focused on the model plant *A. thaliana* and various *Brassica* species (*B. rapa*, *B. nigra*, *B. oleracea*, *B. napus*, *B. juncea* and *B. carinata*). The whole genome sequences of all species except *B. carinata* are available in BRAD (the *Brassica* Database, http://brassicadb.org/brad/downloadOverview.php). In the present study, we identified 52 *MT* genes from various *Brassica* species based on *A. thaliana MT* gene sequences (Table 1). Phylogenetic analysis revealed that all *MT* family genes are closely associated with *AtMTs* (Figure 2), suggesting that they share similar functions or have undergone gene fusion [44]. Of these, *MT1* subfamily genes from *B. nigra*, *B. oleracea*, and *B. juncea* have not been identified, but *BolMT1* and *BjuMT1* have been identified [13]. The number of *MT2* (10) and *MT3* (3) genes in *B. napus* is nearly equal to the sum of these genes in *B. rapa* (5 *MT2* and *1 MT3*) and *B. oleracea* (6 *MT2* and 2 *MT3*), and most genes showed high levels of synteny throughout the genome, reflecting the fact that whole genome duplications and segmental duplications were a major contributor to the expansion of *MTs* during evolution. However, the deduced protein sequence of *BraMT1* in *B. rapa* is longer than the previously published sequence [33], indicating the need for further study to confirm *BraMT1*. In addition, homologs of *BnaMT4A* and *BraMT4*, *BnaMT3A* and *BraMT3*, and *BnaMT1* and *BraMT1* were not detected in the corresponding genomes (Figure 5, Table 1), in accordance with the finding that gene loss typically occurs after polyploidization in eukaryotes [45,46,47]. Furthermore, Cys-rich regions were almost completely conserved among MTs, and the distinct spacer sequences in the Cys-poor linkers were also well-conserved, comprising 7 amino acids in *Brassica* MT1s, 40–42 amino acids in *Brassica* MT2s, 32–34 amino acids in *Brassica* MT3s, and 14–15 amino acids in *Brassica* MT4s (Figure 1). These results are in close agreement with previous predictions that Cys-rich regions will show highly conserved *MT* family genes [13,33], and we infer that the variations in the MTs might be associated with their different functions in plants [48].

To date, plant MTs have been widely characterized, exhibiting different tissue-specific expression patterns [8,14,49,50,51,52]. For example, *AtMT1A* and *AtMT2B* were predominantly expressed in roots and leaves, while *AtMT2A* and *AtMT3* were highly expressed in roots and young leaves [14]. Likewise, notable differences in expression patterns were also found among *MT1*, *MT2A*, and *MT2B* subfamily genes in *B. napus* (Figure 6). *BnaMT1* was expressed at the highest level in siliques of 30D, except for roots and leaves, whereas *B. napus MT2A* genes (with four members; *BnaMT2A*, *BnaMT2C*, *BnaMT2H* and *BnaMT2I*) and *MT2B* genes (with six members; *BnaMT2B*, *BnaMT2D*, *BnaMT2E*, *BnaMT2F*, *BnaMT2G* and *BnaMT2J*; Figure 2) showed variable expression patterns. For example, *BnaMT2A* and *BnaMT2H* were preferentially expressed in buds, and *BnaMT2G* was expressed at low levels in all organs (Figure 6). These differences may be attributed to concentrations and species differences in future works. However, *B. napus MT3* and *MT4* subfamily genes shared similar expression patterns with *AtMT3* and *AtMT4* [14]. *B. napus MT3s* were mainly expressed in stems, roots, and leaves, and *B. napus MT4s* were primarily expressed in developing seeds (Figure 6). The expression patterns of these *BnaMTs* revealed by RT-qPCR corresponded well with the patterns obtained by transcriptome analysis under normal conditions, although there were differences among *B. napus* varieties (Figure 7). Although no comprehensive heavy metal tolerance mechanisms have been uncovered in *Brassica*, distinctive expression patterns were identified among the *B. napus MT* family members in this work, laying the foundation for investigating the biochemical and physiological functions of MTs in plants.

Heavy metal (Cu, Cd, and As) pollution in agricultural soils has become a critical problem affecting crop production and quality. The absorption of these heavy metals by plants plays an important role in the entry of these metals into the food chain [42]. Further, *MTs* have been shown to play an important role in metal homeostasis and tolerance in plants [4,6,9,10,21,26,33,34]. Strikingly, *Brassica* plants exhibit efficient heavy metal uptake and translocation, as well as a high tolerance to heavy metals [31,42], and several *MT* genes in *Brassica* have been reported, especially in Indian mustard (*Brassica juncea* L.) [23,27,33,53]. Recently, three *B. rapa* metallothionein genes (*BrMT1*–*3*) displayed differential expression levels under various exogenous stress factors [54], and MT-like, protein-encoding gene transcription was obviously induced in roots and leaves of *B. napus* under As treatment [55]. Here, we investigated the expression profiles of *BnaMT* family genes in *B. napus* under normal conditions and As^3+^ stress. The *BnaMTs* were obviously induced by the As^3+^ treatments, which is in accordance with findings that MTs are involved in the chelation and sequestration of heavy metals [3,4,55]. Like other plant *MT* genes [4,41,54,55], however, these genes also had different expression profiles in different *B. napus* varieties and in different tissues, with high expression levels in cotyledons and low expression levels in hypocotyls, such as *BnaMT1*, *BnaMT2A*, *BnaMT2B*, *BnaMT2C*, *BnaMT2F*, *BnaMT2J*, *BnaMT3B*, and *BnaMT3C* (Figure 7). These results suggest that hypocotyls might merely be involved in the transport of heavy metal ions, but that these ions accumulate in roots and cotyledons. In addition, *BnaMT3C* was obviously increased in the roots and hypocotyls of B33 and B34, but the higher expression levels of B93 and B113 in cotyledons (Figure 7), which comply with the finding that B33 and B34 exhibited better growth than B93 and B113 under As^3+^ treatment (Figure S2), indicate that they play crucial roles in the response to As^3+^ stress in *B. napus*. Our results provide important information for further functional studies of MT family genes in *B. napus*.

## 4. Materials and Methods

### 4.1. Identification of MT Family Genes in Brassica

The amino acid sequences of MTs from the Arabidopsis Information Resource (TAIR10) database (ftp://ftp.arabidopsis.org) were used as queries for the BLASTp analysis against the whole genome sequences in the *Brassica* database [56]. The candidate sequences with *E*-values ≤ 1 × 10^−20^ were identified and confirmed using the Hidden Markov model (HMM) searches program (HMMER v3.0, http://hmmer.janelia.org/), and the BLAST analysis of the MTs was performed against a *Brassica* protein database constructed using Geneious v4.8.5 software (http://www.geneious.com/, Biomatters, Auckland, New Zealand). The coding sequences (CDS) of the MTs were identified by BLASTn searches against the *Brassica* genome database. The candidate proteins were named using the species abbreviation of the source organism (italicized), the gene family name, and the positions in the subtribe, e.g., *AtMT1B* and *BnaMT1A*. Physicochemical properties, including the molecular weight (kDa), isoelectric point (pI), and the grand average of hydropathy (GRAVY) value of each deduced protein were determined using the online ExPASy-ProtParam tool (http://web.expasy.org/protparam/).

### 4.2. Multiple Sequence Alignment and Phylogenetic Analysis of MTs in Brassica

The deduced amino acid sequences of MT proteins from *A. thaliana* and various *Brassica* species, including *B. rapa*, *B. oleracea*, *B. napus*, *B. juncea*, and *B. nigra*, were subjected to multiple protein sequence alignment using the ClustalW software with default settings [57]. To illustrate the evolutionary relationships of MTs in *Brassica*, a neighbor-joining (NJ) phylogenetic tree was generated with the MEGA v6.0 program (Tokyo Metropolitan University, Tokyo, Japan) using the JTT+I+G substitution model and a bootstrap test with 1000 replicates [58]. The phylogenetic trees were visualized using FigTree v1.4.2 (http://tree.bio.ed.ac.uk/software/figtree/).

### 4.3. Conserved Motif Recognition and Gene Structure Analysis

The CDS of the MTs from the *Brassica* species were retrieved based on their protein sequences, and the corresponding genomic sequences were extracted from the *Brassica* genome sequences. The exon–intron structures of the MTs were analyzed online using the Gene Structure Display Server (GSDS v2.0, http://gsds.cbi.pku.edu.cn/index.php). Conserved motifs were identified using Multipel Expectation Maximization for Motif Elucidation (MEME v4.12.0, http://meme-suite.org/tools/meme) with the following parameters: number of repetitions, any; maximum number of motifs, 15; and optimum width of each motif, between 6 and 300 residues [59]. Each motif with an *E*-value <  1 × 10^−10^ was retained for motif detection.

### 4.4. Chromosomal Locations of MT Family Genes in B. napus

The *MT* family genes were mapped to the rapeseed chromosomes according to their physical distances in the GFF genome files, which were downloaded from the *B. napus* genome database (http://www.genoscope.cns.fr/brassicanapus/) [43]. A map of the chromosomal locations of the MTs was constructed using MapChart v2.0 (https://www.wur.nl/en/show/Mapchart.htm) [60].

### 4.5. Plant Materials and Metal Stress Treatments

*B. napus* seeds were collected from the Rapeseed Engineering Research Center of Southwest University in Chongqing, China (CERCR). Fifty healthy seeds were selected and soaked in a dish (the diameter was 90 mm) containing deionized water for 24 h. Then morphologically uniform seedlings were selected and plugged into a hydroponic system with a float tray (60 cm × 40 cm × 10 cm) for 7 days. Here, the seedlings were exposed to distilled water and 35 μM As^3+^ solutions, respectively. Meanwhile, they were cultivated under long-day conditions (16 h light/8 h dark, 5000 Lux) at 25 °C. After 7 days, the whole roots, hypocotyls, and cotyledons were sampled to analyze the *MT* gene expression patterns; the tissues were snap frozen in liquid nitrogen and stored at −80 °C prior to total RNA extraction. All experiments were repeated three times.

### 4.6. Total RNA Extraction and RT-qPCR Analysis

As the *B. napus* cultivars B33 and B34 grow better than B93 and B113 under heavy metal treatment (Figure S2), they were therefore used for expression analysis. Total RNA was isolated from the samples using a DNAaway RNA Mini-Prep Kit (Sangon Biotech, Shanghai, China). For the tissue-specific expression analysis, RNA was extracted from the roots, hypocotyls, and cotyledons and pretreated with gDNA Eraser (Takara, Dalian, China). Subsequently, 1 μg of the total RNA was used to synthesize first-strand cDNA with an RNA PCR Kit (AMV) Ver. 3.0 (Takara, Dalian, China). The cDNA was subjected to RT-qPCR analysis using SYBR Premix Ex Taq II (Takara, Dalian, China) on a Bio-Rad CFX96 Real Time System (Bio-Rad Laboratories, Hercules, CA, USA) as previously described [61]. *BnACTIN7* (EV116054) was employed as a reference gene to normalize MT gene expression levels via the 2^−ΔΔ*C*t^ method [62]. All experiments were performed with three technical replicates, and the values represent the average ± standard error (SE). The specific primer sequences used in this study were obtained from the qPCR Primer Database [63] and are listed in Supplementary Table S4.

### 4.7. Statistical Analysis

All experiments were repeated three times (three biological replicates). All data were statistically analyzed using the Student’s *t*-test with the statistical analysis software package SPSS v15.0 (IBM Corp, Armonk, NJ, USA).

## 5. Conclusions

In this study, we identified 60 *MTs* from *A. thaliana* and five *Brassica* species. The phylogenetic analysis showed that all *MT* family genes are closely associated with the *AtMTs*. Genome-mapping analysis revealed high levels of synteny throughout the genome due to whole genome duplication and segmental duplication events. In addition, all 16 *BnaMTs* were induced by heavy metal stress, especially in cotyledons versus roots and hypocotyls. Finally, *BnaMT3C* might improve the response to As^3+^ stress in *B. napus*. Our results provide a basis for the further functional analysis of the molecular functions of *MT* family genes in *B. napus*.

## Figures and Tables

**Table 1:** List of *Metallothionein* (*MT*) genes identified in the *A. thaliana* and *Brassica* genomes.

Groups	Name	Gene ID	Chr.	Start (bp)	End (bp)	Length (bp)	Length (aa)	MW (KDa)	pIs	Exon	Intron
*MT1*	*AtMT1*	AT1G07600	AtChr1	2338904	2339321	138	45	4.580	4.23	2	1
	*AtMT1C*	AT1G07610	AtChr1	2341542	2342123	138	45	4.495	4.54	2	1
	*AtMT1B*	AT5G56795	AtChr5	22972042	22972449	156	51	5.428	10.25	2	1
	*BraMT1*	Bra015594	BraA10	766670	772706	450	149	16.77	9.32	4	3
	*BnaMT1*	BnaA10g04950D	BnaA10	2673266	2673770	138	45	4.480	3.92	2	1
	*BolMT1*	DK501359	UN	UN	UN	138	45	4.412	3.92	UN	UN
	*BjuMT1*	EF471214	UN	UN	UN	138	45	4.439	3.92	UN	UN
*MT2*	*AtMT2A*	AT3G09390	AtChr3	2889486	2890229	246	81	8.163	4.35	2	1
	*AtMT2B*	AT5G02380	AtChr5	506498	507244	234	77	7.766	4.54	2	1
	*BraMT2A*	Bra001309	BraA03	15803598	15803933	246	81	8.197	4.17	2	1
	*BraMT2B*	Bra005720	BraA03	275453	275766	243	80	8.033	4.29	2	1
	*BraMT2C*	Bra009595	BraA10	16182058	16182453	243	80	8.031	4.29	2	1
	*BraMT2D*	Bra028875	BraA02	269835	270,238	246	81	8.386	4.20	2	1
	*BraMT2E*	Bra029765	BraA05	23082833	23083178	243	80	8.024	4.35	2	1
	*BolMT2A*	Bol000591	Scaffold000521	37950	38342	243	80	8.031	4.29	2	1
	*BolMT2B*	Bol011307	Scaffold000212	445183	445551	243	80	8.054	4.58	2	1
	*BolMT2C*	Bol012825	BolC02	305134	305536	246	81	8.386	4.20	2	1
	*BolMT2D*	Bol015273	BolC03	101213	101535	243	80	8.137	4.29	2	1
	*BolMT2E*	Bol023080	BolC01	37533909	37534079	171	56	5.920	4.15	1	0
	*BolMT2F*	Bol033925	Scaffold000040	316614	316949	246	81	8.147	4.15	2	1
	*BnaMT2A*	BnaA03g30680D	BnaA03	14857530	14858170	240	79	7.966	3.81	2	1
	*BnaMT2B*	BnaA03g54880D	A03_random	44751	45330	243	80	8.077	4.29	2	1
	*BnaMT2C*	BnaA05g29010D	BnaA05	20416060	20416808	243	80	8.024	4.35	2	1
	*BnaMT2D*	BnaA10g27170D	BnaA10	17170773	17171571	243	80	8.031	4.29	2	1
	*BnaMT2E*	BnaAnng00330D	Ann_random	321245	322046	246	81	8.370	4.20	2	1
	*BnaMT2F*	BnaC02g03550D	BnaC02	1685618	1686330	246	81	8.386	4.20	2	1
	*BnaMT2G*	BnaC03g00710D	BnaC03	346945	347282	216	71	7.284	4.08	2	1
	*BnaMT2H*	BnaC03g35960D	BnaC03	21778025	21778740	240	79	7.950	3.79	2	1
	*BnaMT2I*	BnaC05g43490D	BnaC05	40327486	40328304	243	80	8.028	4.58	2	1
	*BnaMT2J*	BnaCnng40400D	Cnn_random	38972401	38973067	243	80	8.031	4.29	2	1
	*BjuMT2A*	BjuA008858	BjuA03	321697	322020	243	80	8.033	4.29	2	1
	*BjuMT2B*	BjuA020647	BjuA05	29317680	29319366	312	103	10.661	4.24	3	2
	*BjuMT2C*	BjuA040818	BjuA02	430697	431097	246	81	8.370	4.20	2	1
	*BjuMT2D*	BjuA044587	BjuA10	19619066	19619458	243	80	8.031	4.29	2	1
	*BjuMT2E*	BjuB001621	BjuB08	169755	170152	243	80	8.059	4.29	2	1
	*BjuMT2F*	BjuB005939	BjuB01	43381550	43383641	297	98	10.057	3.93	3	2
	*BjuMT2G*	BjuB012072	BjuB05	20387272	20387699	246	81	8.353	4.11	2	1
	*BjuMT2H*	BjuB031838	BjuB03	12766757	12767103	222	73	7.596	4.35	2	1
	*BjuMT2I*	BjuB044439	BjuB02	52757313	52757655	243	80	8.061	4.29	2	1
	*BniMT2A*	BniB001954-PA	BniB08	30524166	30524483	243	80	8.075	4.29	2	1
	*BniMT2B*	BniB007929-PA	BniB05	2189985	2190357	243	80	7.978	4.35	2	1
	*BniMT2C*	BniB023579-PA	BniB02	28213025	28213453	246	81	8.353	4.11	2	1
	*BniMT2D*	BniB039464-PA	BniB07	32240383	32240733	222	73	7.596	4.35	2	1
	*BniMT2E*	BniB045064-PA	BniB03	44318189	44318591	225	74	7.569	4.08	2	1
*MT3*	*AtMT3*	AT3G15353	AtChr3	5180642	5181586	210	69	7.373	4.35	3	2
	*BraMT3*	Bra027254	BraA05	20683494	20683920	204	67	7.183	4.17	3	2
	*BolMT3A*	Bol011145	BolC05	28120530	28120940	204	67	7.158	4.15	3	2
	*BolMT3B*	Bol025753	BolC03	17061982	17062438	198	65	7.016	4.40	3	2
	*BnaMT3A*	BnaA05g24200D	BnaA05	18177871	18178669	204	67	7.127	4.15	3	2
	*BnaMT3B*	BnaC03g39060D	BnaC03	24091592	24092154	198	65	7.016	4.40	3	2
	*BnaMT3C*	BnaC05g38240D	BnaC05	37017131	37017881	204	67	7.158	4.15	3	2
	*BjuMT3*	BjuB025665	BjuB01	40434253	40434691	204	67	7.187	4.15	3	2
	*BniMT3*	BniB008959-PA	BniB05	4957466	4957899	204	67	7.187	4.15	3	2
*MT4*	*AtMT4B*	AT2G23240	AtChr2	9895855	9896325	261	86	8.437	5.58	2	1
	*AtMT4A*	AT2G42000	AtChr2	17529243	17530443	366	121	12.229	7.62	4	3
	*BraMT4*	Bra000590	BraA03	11951235	11951571	261	86	8.480	7.37	2	1
	*BnaMT4A*	BnaA03g23200D	BnaA03	11067719	11068213	261	86	8.480	7.37	2	1
	*BnaMT4B*	BnaC03g27400D	BnaC03	15895032	15895647	261	86	8.468	6.97	2	1
	*BjuMT4*	BjuO006263	Contig407_1_341981	122112	122451	261	86	8.500	6.97	2	1
	*BniMT4*	BniB049568-PA	BniB03	29749524	29749863	261	86	8.472	6.97	2	1

Note, At, *A. thalinana*; Bra, *B. rapa*; Bol, *B. oleracea*; Bni, *B. nigra*; Bna, *B. napus*; and Bju, *B. juncea*; Chr., Chromosome; UN, unknown.

## Supplementary Materials

Supplementary materials can be found at http://www.mdpi.com/1422-0067/19/8/2181/s1.
