# Supplementary figures and images for: Genome-Wide Characterization and Analysis of Metallothionein Family Genes That Function in Metal Stress Tolerance in Brassica napus L
Source: Int J Mol Sci. 2018 Jul 26;19(8):2181. doi: 10.3390/ijms19082181 (PMC6121329; doi:10.3390/ijms19082181)

B33-CK

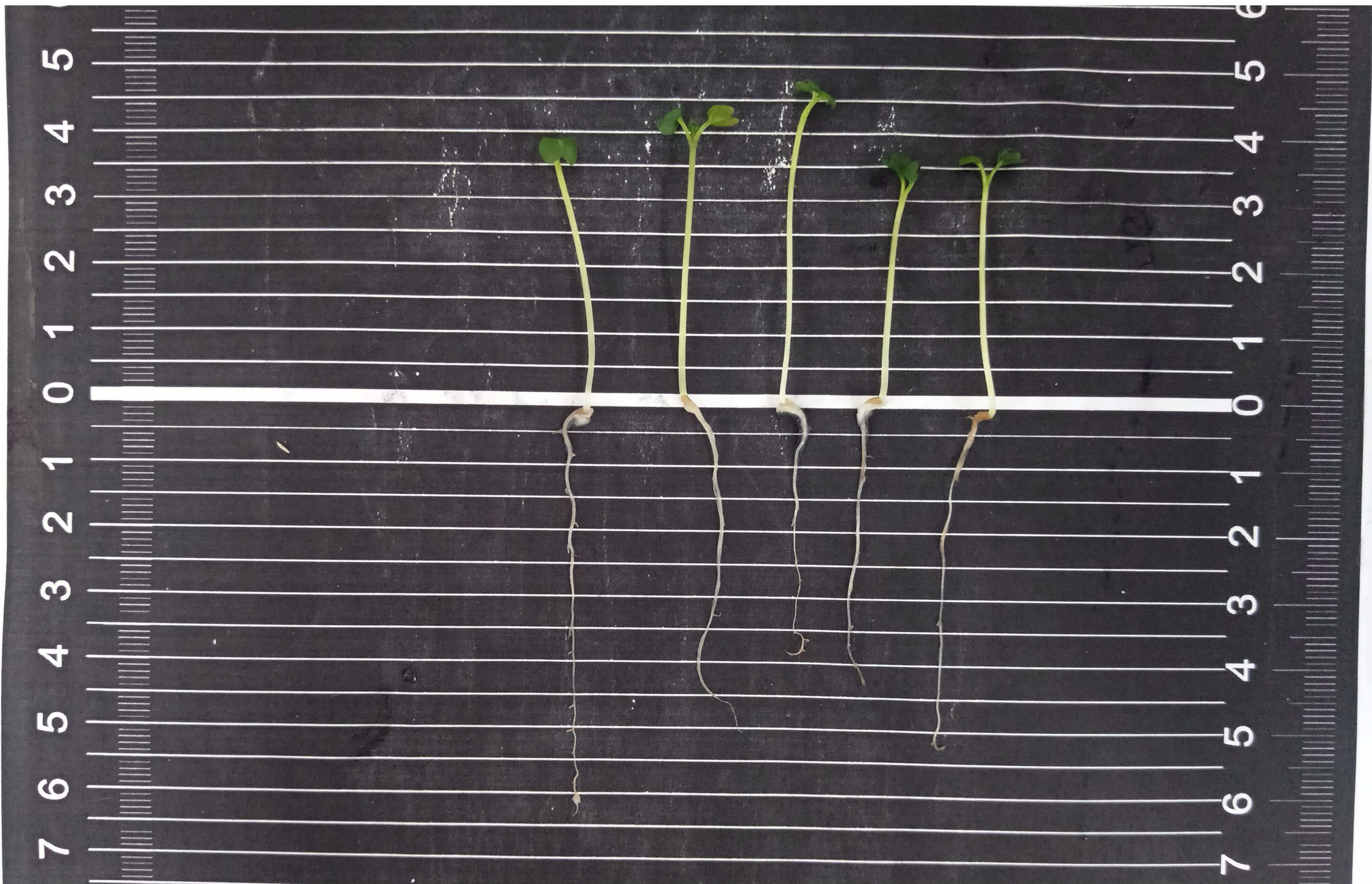

B33-As<sup>3+</sup>

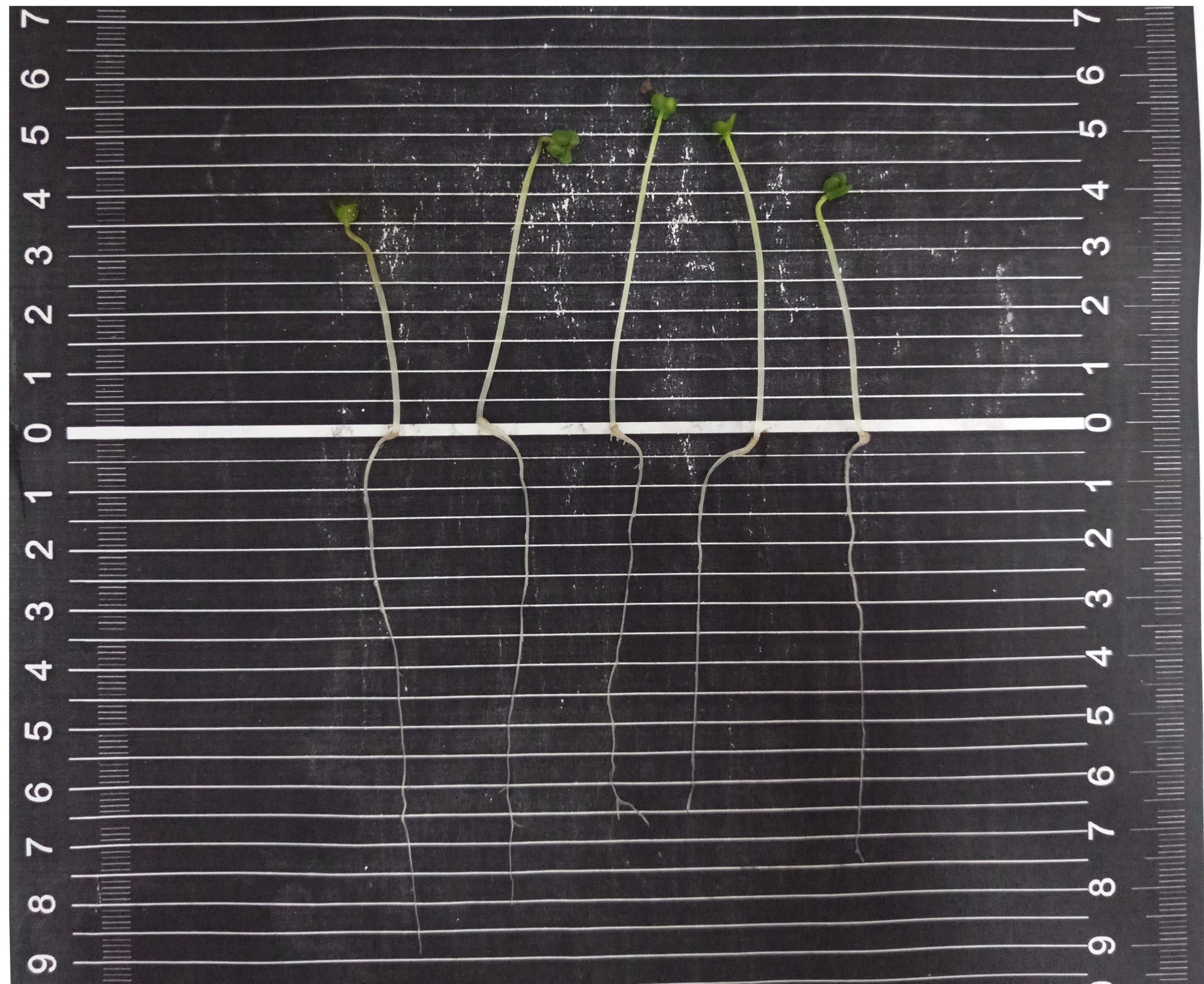

B34-CK

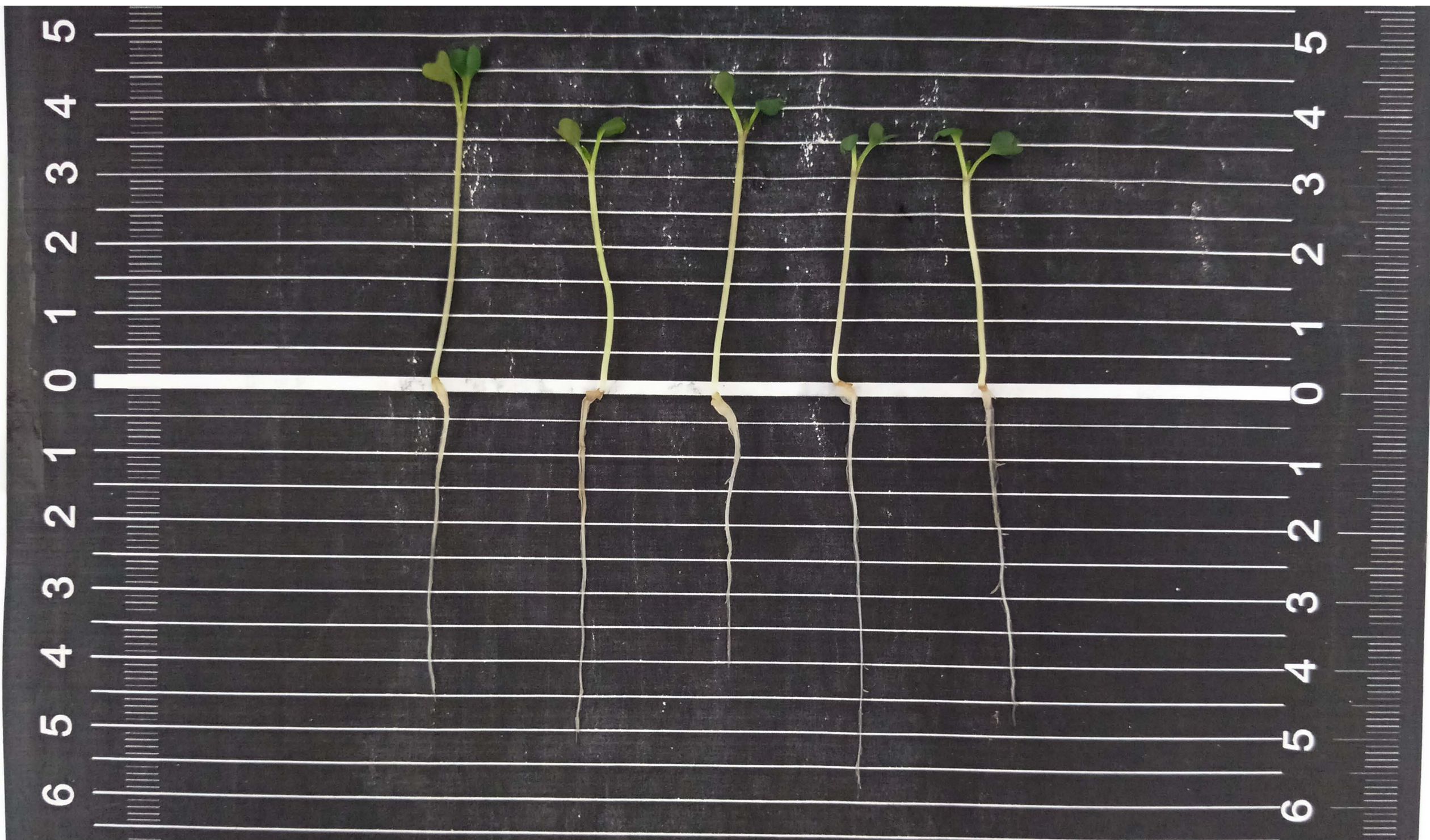

B34-As<sup>3+</sup>

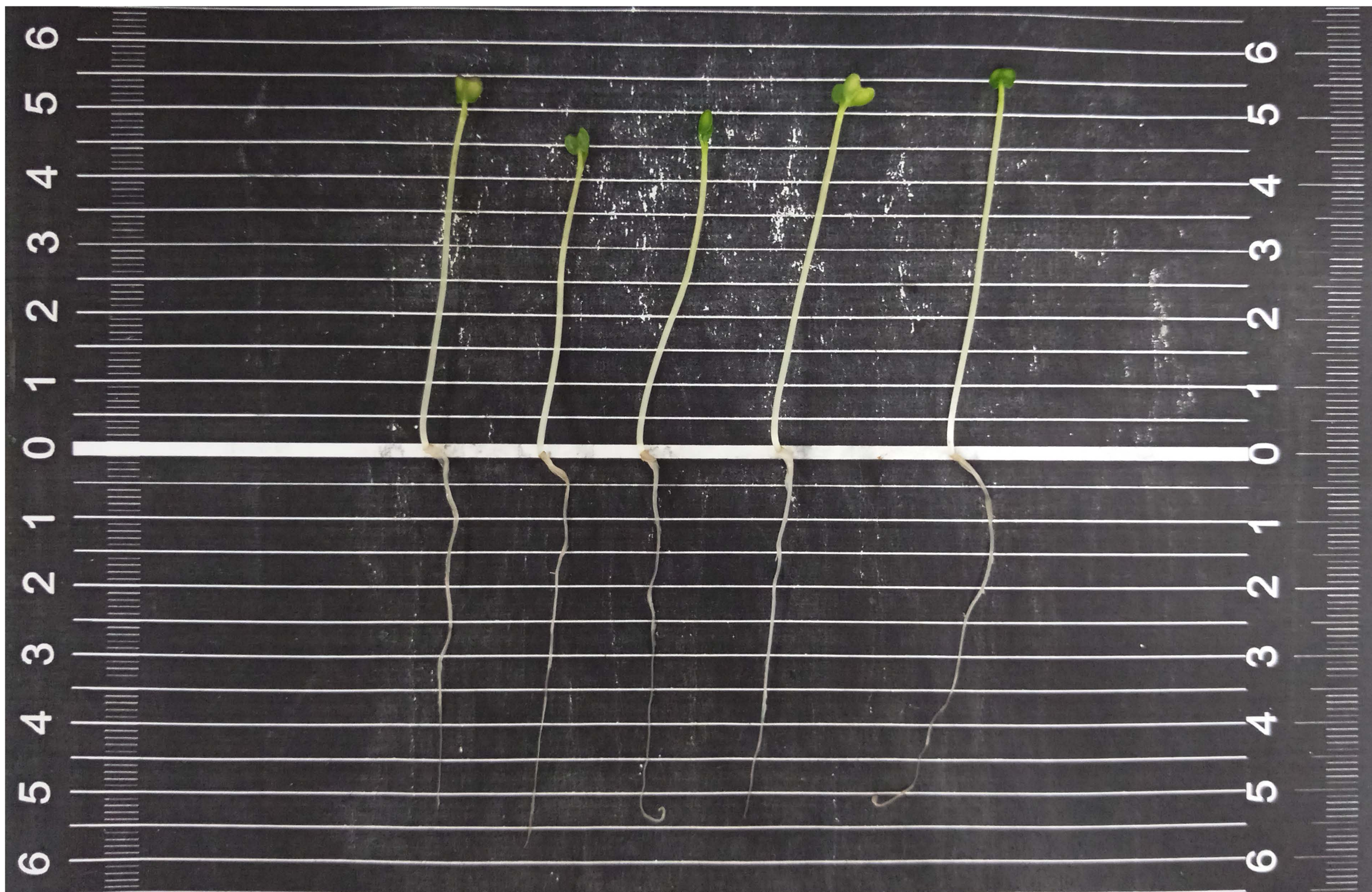

B93-CK

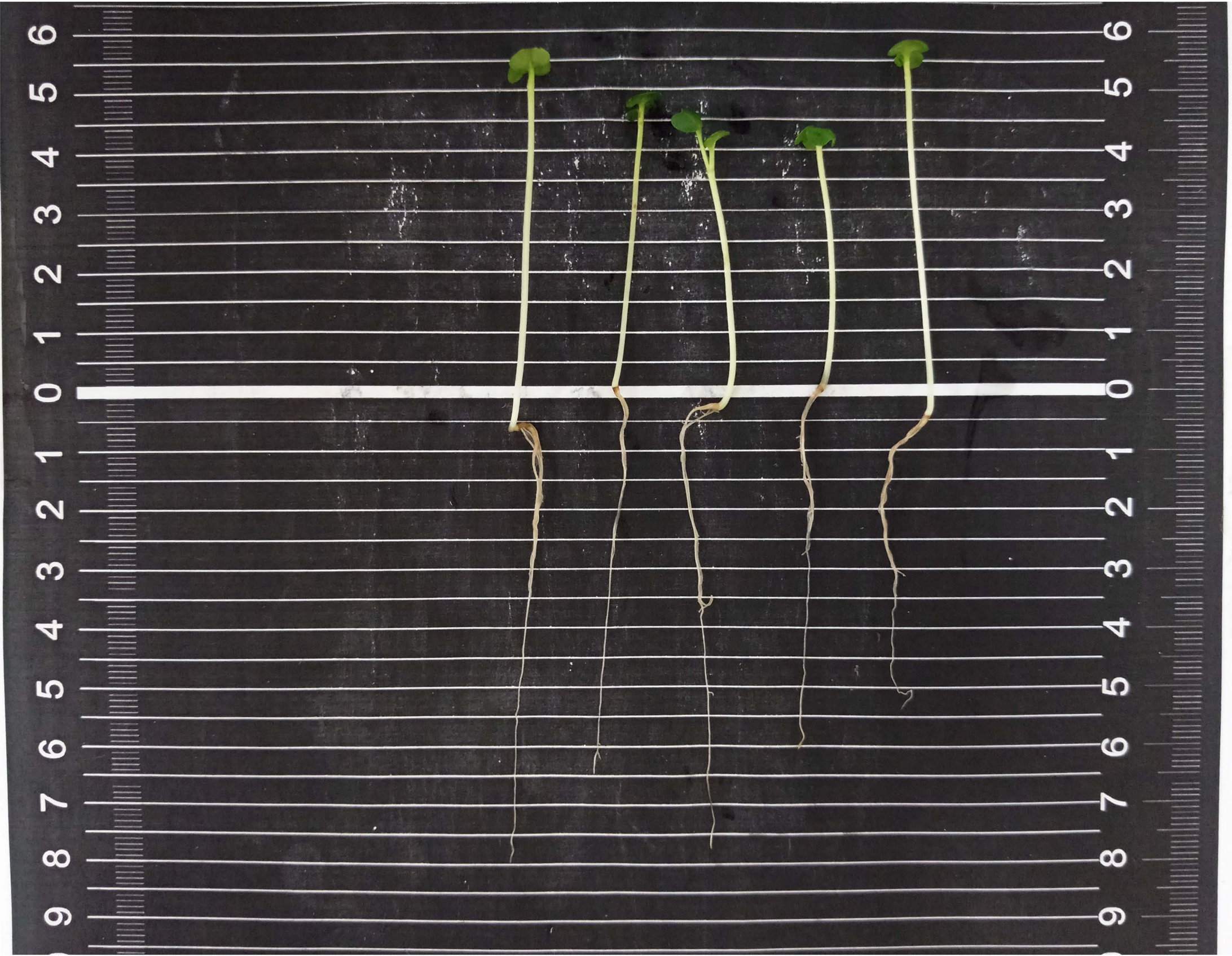

B93-As<sup>3+</sup>

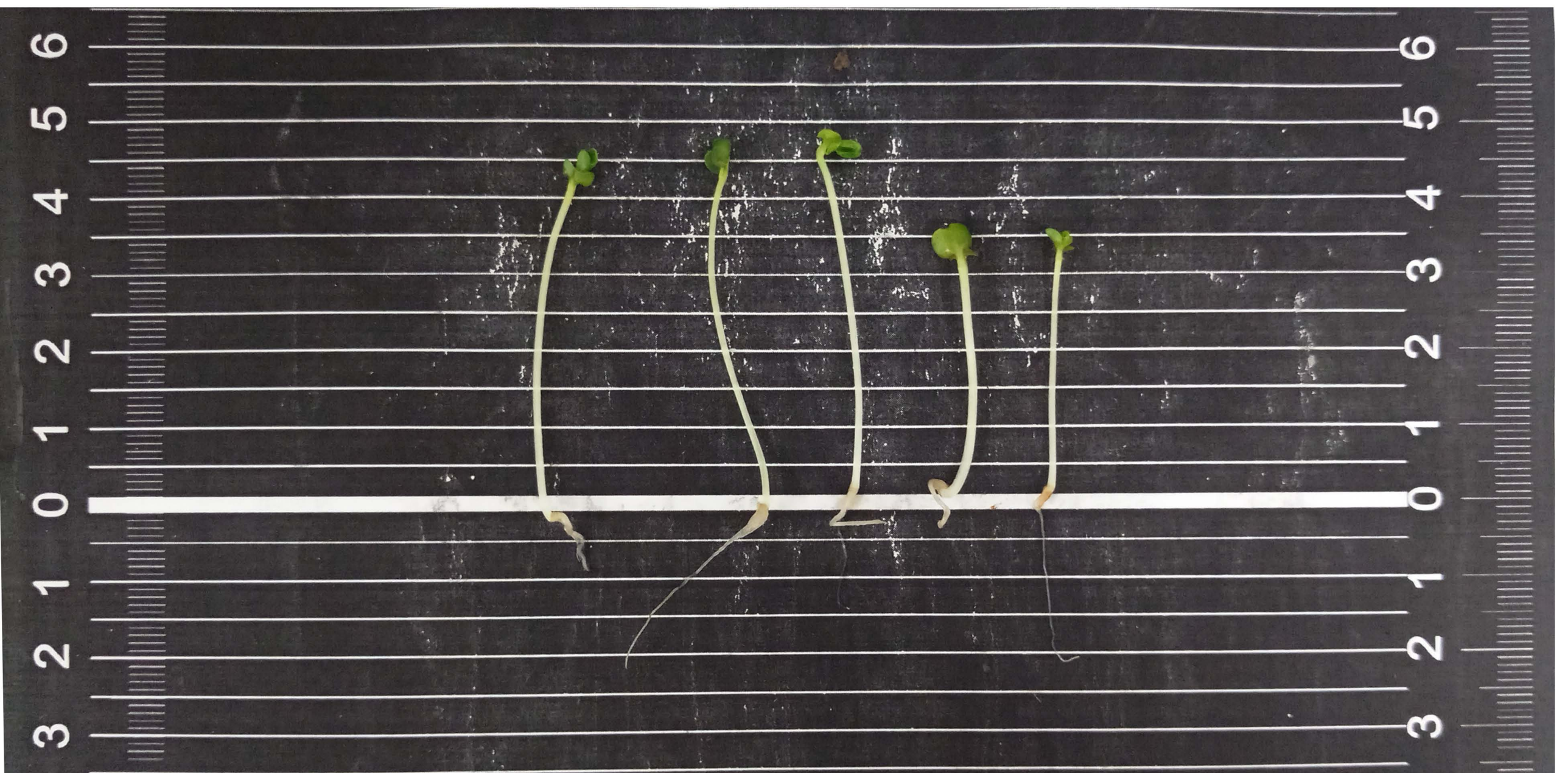

B113-CK

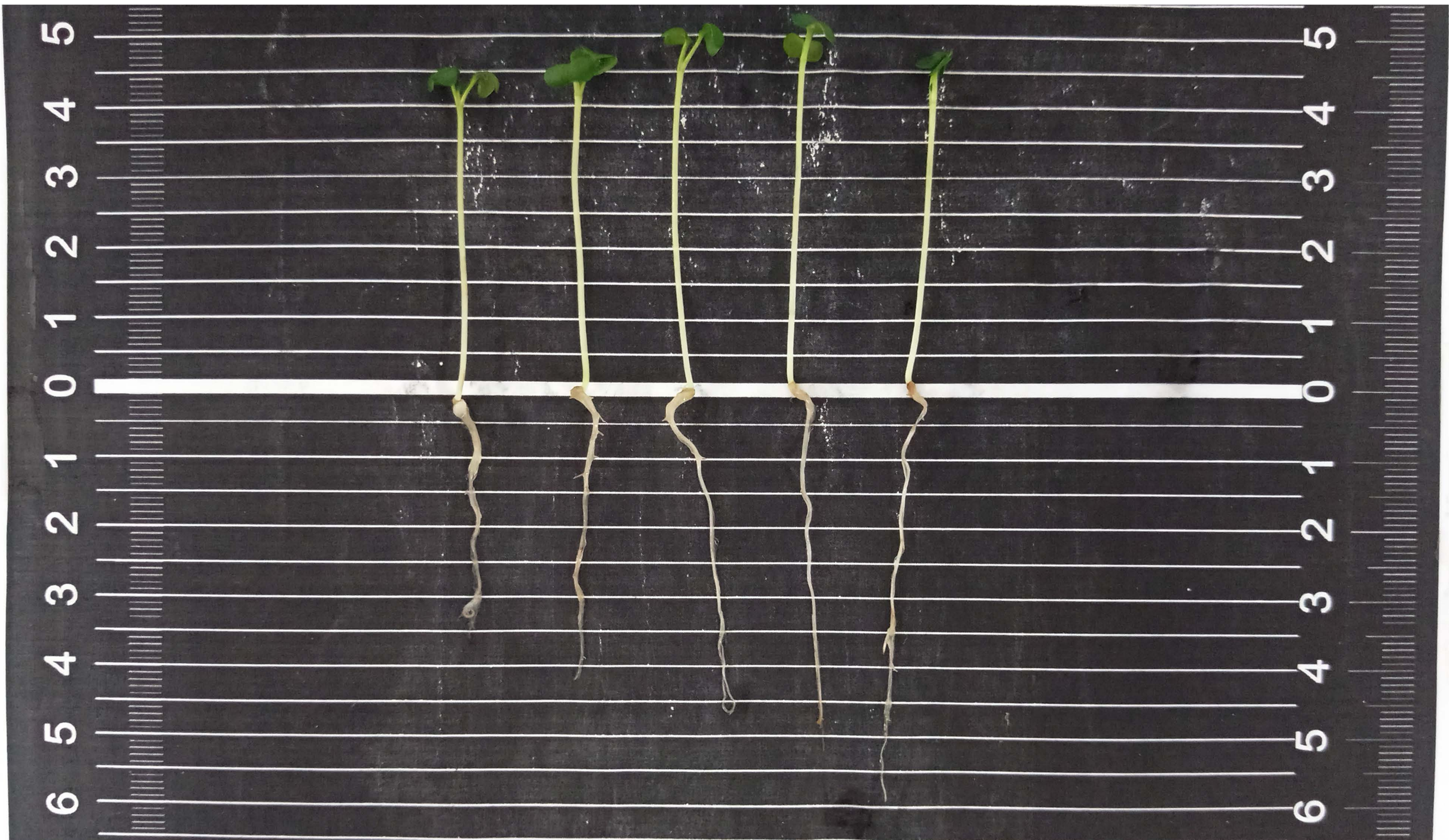

B113-As<sup>3+</sup>

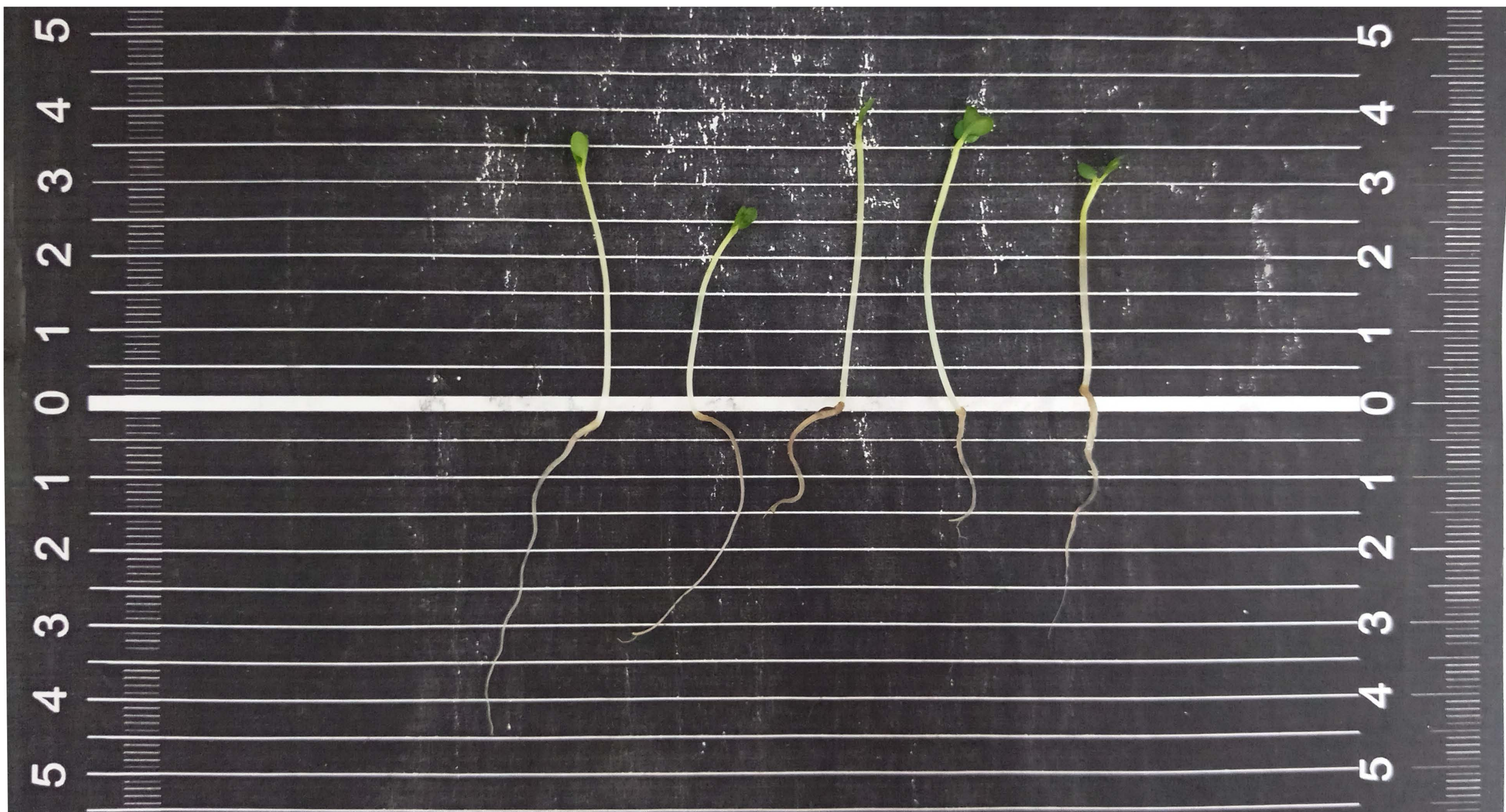

Supplement: Supplementary file 1 [file ijms-19-02181-s001.zip › Figure S2. The situations of Brassica napus B33, B34, B93 and B113 under the control and As3+ treatment.pdf]

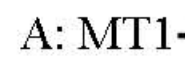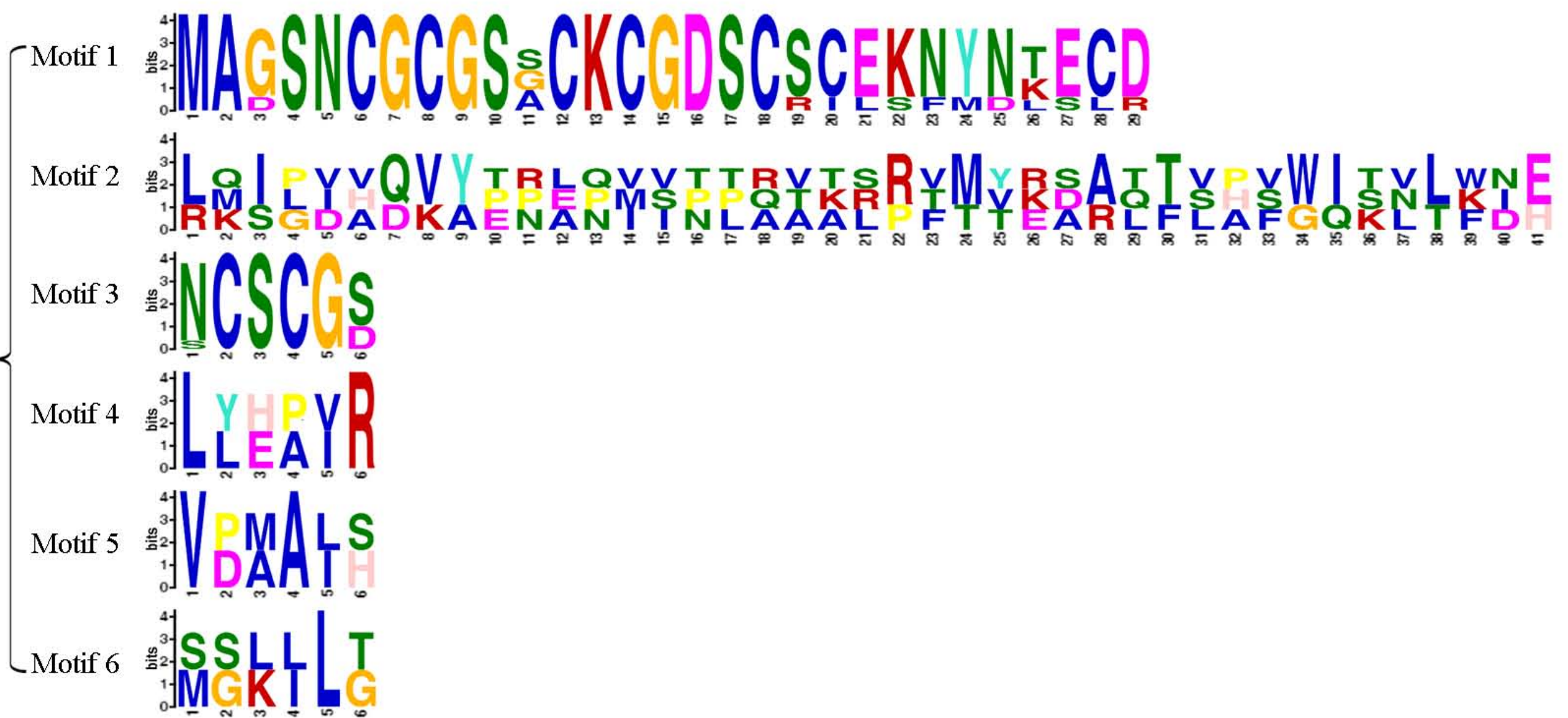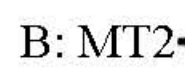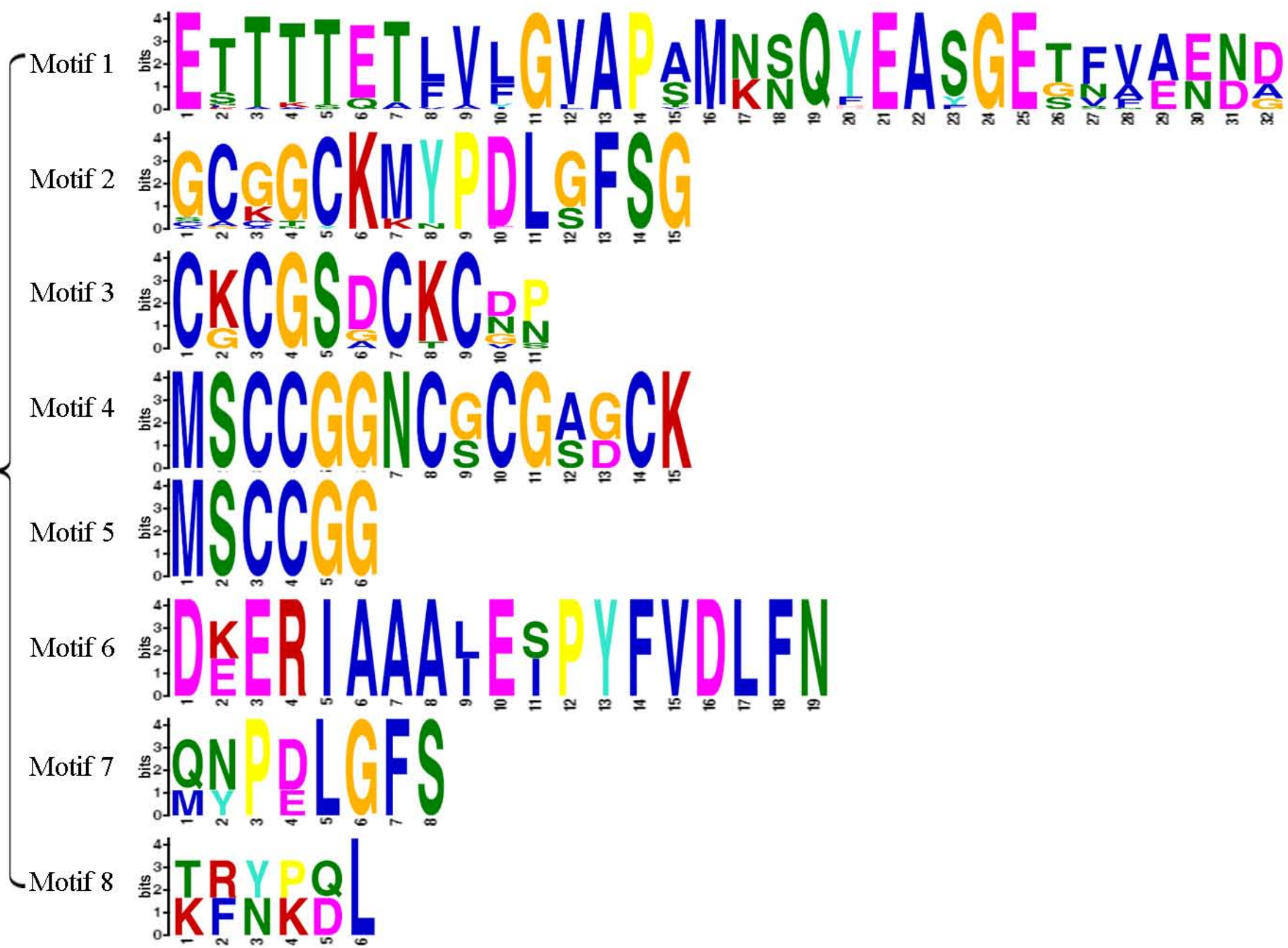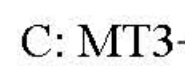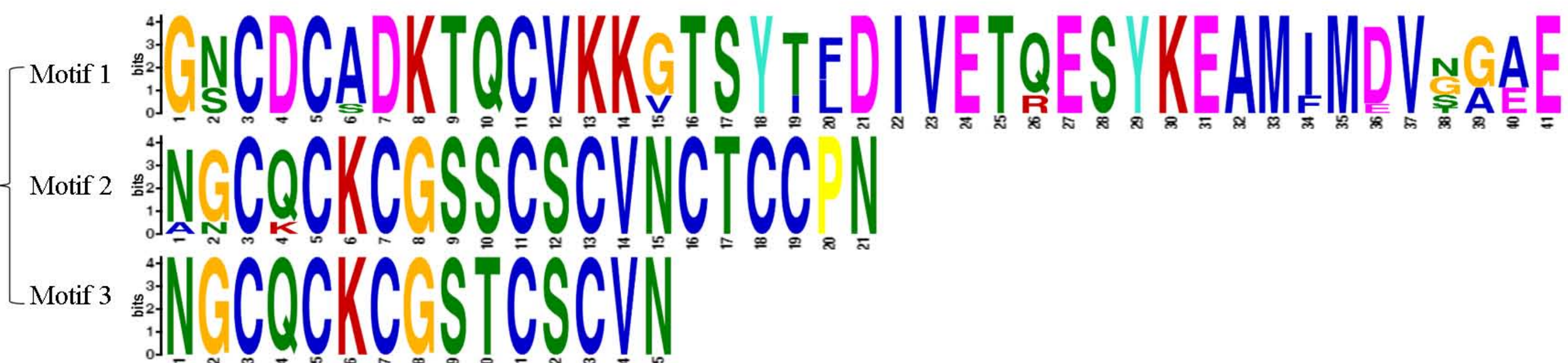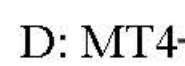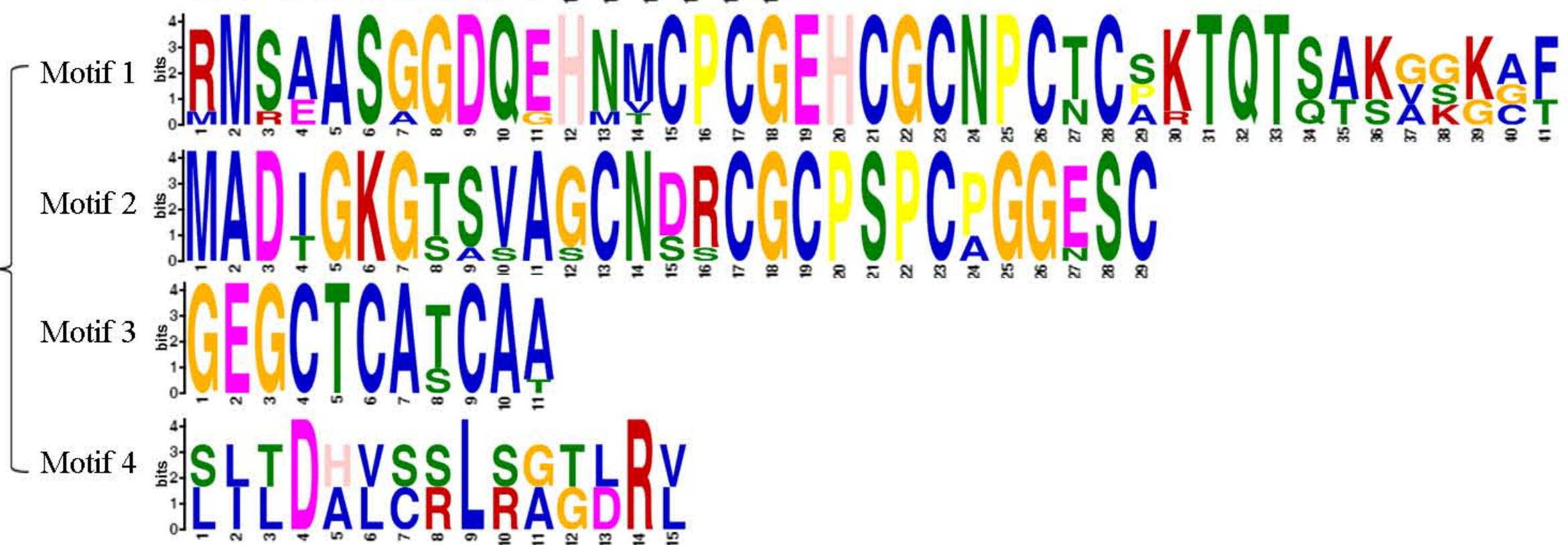

Supplement: Supplementary file 1 [file ijms-19-02181-s001.zip › Figure S1. Sequence logos of the motifs in MT proteins.pdf]
